# Extra-abdominal desmoid tumor fibromatosis: a multicenter EMSOS study

**DOI:** 10.1186/s12885-021-08189-6

**Published:** 2021-04-20

**Authors:** Pierluigi Cuomo, Guido Scoccianti, Alberto Schiavo, Valentina Tortolini, Catrin Wigley, Francesco Muratori, Davide Matera, Mariia Kukushkina, Philipp Theodor Funovics, Marie-Theres Lingitz, Reinhard Windhager, Sander Dijkstra, Jorrit Jasper, Daniel A. Müller, Dominik Kaiser, Tamás Perlaky, Andreas Leithner, Maria Anna Smolle, Domenico Andrea Campanacci

**Affiliations:** 1 Orthopaedic Oncology Department, Azienda Ospedaliero Universitaria Careggi, Florence, Italy; 2 Sarcoma Unit, Royal National Orthopaedic Hospital, Stanmore, UK; 3 CERM Foundation, University of Florence, Florence, Italy; 4 Department of Skin and Soft Tissue Tumors, National Cancer Institute, Kiev, Ukraine; 5 Department of Orthopaedics and Trauma Surgery, Medical University of Vienna, Vienna, Austria; 6 Department of Orthopaedic Surgery, University of Leiden, Leiden, The Netherlands; 7 Balgrist University Hospital, Zurich, Switzerland; 8 Department of Orthopaedics, Semmelweis University, Budapest, Hungary; 9 Department of Orthopaedics and Trauma, Medical University of Graz, Graz, Austria

## Abstract

**Background:**

Extra-abdominal desmoid tumor fibromatosis (DTF) is a rare, locally aggressive soft tissue tumour. The best treatment modality for this patient cohort is still object of debate.

**Questions/purpose:**

This paper aimed to (1) to compare the outcomes of DTF after different treatment modalities, (2) to assess prognostic factors for recurrence following surgical excision, and (3) to assess prognostic factors for progression during observation.

**Methods:**

This was a retrospective multicenter study under the patronage of the European Musculoskeletal Oncology Society (EMSOS). All seven centres involved were tertiary referral centres for soft tissue tumours. Baseline demographic data was collected for all patients as well as data on the diagnosis, tumour characteristics, clinical features, treatment modalities and whether they had any predisposing factors for DTF.

**Results:**

Three hundred eighty-eight patients (240 female, 140 male) with a mean age of 37.6 (±18.8 SD, range: 3–85) were included in the study. Two hundred fifty-seven patients (66%) underwent surgical excision of ADF, 70 patients (18%) were observed without therapy, the residual patients had different conservative treatments. There were no significant differences in terms of tumour recurrence or progression between the different treatment groups. After surgical excision, younger age, recurrent disease and larger tumour size were risk factors for recurrence, while tumours around the shoulder girdle and painful lesions were at risk of progression in the observational group.

**Conclusion:**

Local recurrence rate after surgery was similar to progression rates under observation. Hence, observation in DTF seems to be justified, considering surgery in case of dimensional progression in 2 consecutive controls (3 and 6 months) and in painful lesions, with particular attention to lesions around the shoulder girdle.

## Introduction

Desmoid tumor fibromatosis (DTF) is a rare, soft tissue tumor originating from the clonal proliferation of spindle cells [1, 2]. The incidence is three in every 3.5 million and has a 2:1 female: male predisposition [3–6]. DTF occurs due to a mutation in the gene encoding of the βcatenin in sporadic cases, or the APC genes in familial cases, which are usually associated with familial adenomatous polyposis syndrome [1, 7–9].

Sporadic cases are typically characterized by locally aggressive disease, without metastatic potential, and most commonly located in the limbs, girdle, trunk or the neck [10–13]. Conversely, familial cases, are classically located intra-abdominally involving the mesentery and/or intestinal wall [4, 5, 14].

Despite being uncommon, benign and void of metastatic potential, its aggressive nature and high tendency for local recurrence following excision makes DTF an area of active debate within the literature [11, 13, 15]. Local recurrence rates have been reported between 15 and 77% at an average recurrence periods around 14.1 months [16–18]. Up to two thirds of surgically excised lesions have been reported to recur, independent of resection margins due to the infiltrative nature of DTF, which often threatens local structures such as neurovascular bundles or neighboring parenchyma [17, 19–28]. Studies investigating alternative therapies such as radiotherapy, cryotherapy, isolated limb perfusion as well as traditional pharmacological interventions have all proven to confer no outcome advantage for primary disease [9, 29–35], although promising results have been recently reported on kinase inhibitors particularly for advanced or recurrent disease [36].

The underwhelming results of these current treatment options, as well as the ability of DTF to spontaneously regress have led to an increasing trend towards conservative management (i.e. regular 3, 6, 12 months regular interval clinical and instrumented assessment) [10, 29] and in 2018 the Desmoid Tumor Working group agreed that such an “active surveillance” should be the first line of management for desmoid tumors [37].

Retrospective studies have previously aimed at identifying risk factors for local recurrence after surgery or for disease progression after observation [3, 6, 10, 24, 38]. Patients younger than 35 years, female gender and a maximum diameter larger than 5 cm have been found by most to be negative prognostic indicators following surgical excision [29, 39, 40]. Resection margins on the other hand have had conflicting evidence for their significance [16, 23, 26, 41]. Studies adopting conservative management and aiming at identifying risk factors for disease progression have also failed to deduce any convincing conclusions.

Given this uncertainty of a generally agreed treatment standard, the aims of this multicentric study under the patronage of the European Musculoskeletal Oncology Society (EMSOS) using data from tertiary soft tissue tumors referral centers therefore, were (1) to compare the outcomes of DTF after different treatment modalities, (2) to assess prognostic factors for recurrence following surgical excision, and (3) to assess prognostic factors for progression during observation. The null hypothesis was that there is no difference between surgical and non-surgical management of DTF.

## Materials and methods

A proposing Institution submitted a study project to the EMSOS to investigate DTF treatment modalities. An electronic database was designed to collect data from the multiple centers involved. The study was approved by the EMSOS Board and advertised via its two international conferences (2018 and 2019) and emailing list. Study participation was voluntary and open to any center specializing in the management of soft tissue tumors. A minimum contribution of 30 patients per center was required for study inclusion. Data from patients with histologically diagnosed DTF could be included. Each center was responsible of obtaining local ethic committee approval.

A Microsoft Excel datasheet was designed by a selection of the lead authors. This was available to download from the EMSOS website (www.emsos.org). Data entries were grouped into the following eight categories.

1. Identification: affiliation ID, patient ID (anonymous, numeric)
2. Demographics: age at diagnosis, gender
3. Diagnosis: presentation (primary; recurrent), symptoms onset to diagnosis delay interval, biopsy (needle; incisional), beta catenin (positive; negative; non investigated)
4. Tumor extension: site (shoulder girdle; arm; forearm; hand/wrist; pelvic girdle; thigh; leg; ankle; foot), size (< 5 cm; 5–10 cm; > 10 cm), depth (extra-fascial; deep), nerve involvement (yes; no)
5. Possible predisposing factors: previous local events (trauma; surgery; none), hormonal status (pregnancy; recent pregnancy; menopause; none)
6. Clinical features: pain (absent; mild; moderate; severe), neurologic symptoms (yes; no)
7. Treatment: first treatment (active surveillance; surgery; NSAIDS; hormone therapy; chemotherapy; tyrosine kinase inhibitors; radiotherapy), margins (R0; R1; R2; not applicable), duration of medical treatment (not applicable; < 6 months; 6–12 months; 12–24 months; ongoing), outcome (remission; stable disease; progression/recurrence)
8. Follow-up: latest follow-up (months), follow-up protocol (clinical only; clinical & ultrasound; clinical & MRI), local status (NED; stable; progressing).

### Statistical considerations and data analysis

Statistical analysis was performed using R software (R Core Team 2013 and conducted by a senior statistician (V.T.). An independent chi square test was performed to compare the outcome between treatment groups. To investigate prognostic factors, multiple logistic regression analysis was run for the following independent variables: age, gender, presentation, site, pain, diagnostic delay, size, depth, margins.

(1) To compare outcomes between different treatment options the rates of recurrences after surgical treatment or progression after non-surgical management were compared between the different patient groups. (2) To assess prognostic factors of local recurrence rates after surgical excision, local recurrence was defined as documented instrumental presence of disease assessed with ultrasound or MRI (3) Finally, to assess factors influencing disease progression under observation, progression was defined as an increase in tumor size which required a shift in DTF management.

## Results

Seven European Institutions affiliated with EMSOS applied to take part to the project and were included in the study. The seven institutions represented six countries; Italy, Hungary, Netherlands, Ukraine, Austria and Switzerland. Three hundred and eighty-eight patients with a histologically diagnosed DTF were recruited. There were 240 female and 148 males. Mean age at diagnosis was 37.6 (±18.8 SD, range: 3–85) years old. A primary disease was diagnosed in 296 of the patients on average at 13.8 (±20.3 SD, range: 0–120) months after the onset of symptoms. Histologic diagnosis was achieved through an incisional biopsy in a slightly higher number of patients (52.7%) than those who received a needle biopsy. Beta-catenin mutation was investigated in 123 patients (31%) and was positive in the vast majority of them (91%).

In almost one third of the patients (32.3%) lesions were located at the shoulder girdle. The second most frequently involved site was the thigh (19.8%) followed by the pelvic girdle (19,5%). Other less frequent sites included arm, leg, forearm, foot and hand/wrist which were involved in 10.0, 9.5, 4.8, 2.9 and 0.8% of the cases, respectively. Almost half of the lesions (46.8%) had a maximum diameter between 5 and 10 cm large. As for the remaining cases, 29.5% of the lesions were smaller than 5 cm and 23.5% were larger than 10 cm. The vast majority of the lesions (86.4%) were deep lesions located underneath fascia. In 18.3% of the cases nerve involvement was identified on imaging, and of this cohort, 67.1% reported neurological symptoms. Pain was reported in 62.2% of patients, which was graded as mild, moderate or severe in 55.9, 31.9 and 8.1% of the patients retrospectively.

Nine percent of the patients had a history of previous local injury, whilst 18.3% had received surgery at the anatomical location of the DTF. Only 1% of ADF cases occurred during pregnancy, and 2.9% during puerperium.

Demographics and pre-treatment clinical data are summarized in Table 1 according to the received treatment.

**Table 1:** Patients demographics and clinical data according to treatment Group

Group	I: Chemotherapy (#17)	II: Hormone therapy (#15)	III: NSAIDS (#16)	IV: Radiotherapy (#13)	V: Surgery (#257)	VI: Active surveillance (#70)	Overall (#388)
Age (years)							
Mean	30,8	39,7	38,1	45,8	36,6	40,6	37,5
Min	10	15	11	15	3	10	3
Max	53	74	63	69	81	85	85
Gender							
Female	10	11	8	7	158	46	240
Male	7	4	8	6	99	24	148
Presentation							
Primary	11	10	14	11	193	57	296
Recurrent	6	5	2	2	62	13	90
Diagnosis delay							
Mean	20,9	15,8	15,2	13,5	13,1	13,5	13,8
Min	1	1	2	1	0	0	0
Max	72	72	53	60	120	120	120
Biopsy							
Incisional	15	10	3	1	134	27	190
Needle	2	5	13	11	98	41	170
Beta catenin							
Not invastigated	13	9	5	2	179	39	247
Negative			1		7	3	11
Positive	4	6	10	10	57	25	112
Location							
Arm		2	1		25	9	37
Foot	1				8	2	11
Forearm	2				12	4	18
Hand/wrist					3		3
Leg	2	2	2	3	23	3	35
Pelvic girdle	5	5	4		41	17	72
Shoulder girdle	3	5	7	7	78	19	119
Thigh	4		1	3	53	12	73
Size							
< 5 cm	3	3	5		78	24	113
5–10 cm	7	9	5	9	118	32	180
> 10 cm	7	3	6	4	56	13	89
Location							
Deep	17	15	15	12	208	57	324
Extra-fascial			1	1	38	11	51
Nerve Involvement							
Present	4	3	3	8	40	9	67
Neurologic symptoms							
Present	3	4	2	4	28	4	45
Previous local events							
Surgery	6	5	1	1	38	17	68
Trauma	2	1	3	1	22	4	33
Hormonal status (females only)							
Menopause	1		3		22	7	33
Pregnancy	1				2	1	4
Recent pregnancy	1			1	5	4	11
Pain							
Absent	3	3	4	3	97	25	135
Mild	9	4	5	7	74	34	133
Moderate	4	7	6		45	9	71
Severe	1	1	1	3	11	1	18

Two hundred fifty-seven patients (66.2%) underwent surgical excision of DTF (Group I). Resection margin data was available for 233 patients and showed that R0, R1 and R2 margins were obtained in 40.3, 39.9 and 19.7% respectively.

Forty-eight patients were administered medical treatment consisting of chemotherapy (17, Group II), hormone therapy (15, Group III) or NSAIDS (16, Group IV). All the patients in Group II received methotrexate and vinblastine. Thirteen patients, (Group V), received radiotherapy. The remaining 70 patients, (Group VI), underwent conservative management with active clinical and instrumental observation (active surveillance).

### Outcome of different treatment groups

Outcome data for 375 of the 388 patients was available and is summarized in Table 2.

**Table 2:** Outcome different DTF treatments

	Outcome		
	Remission	Stable disease	Progression
**Chemotherapy**	2	13	2
**Hormone therapy**	1	8	6
**NSAIDS**	2	9	5
**Radiotherapy**	5	7	1
**Surgery**	146	11	91
**Active surveillance**	8	32	27

In Group I, after a mean follow-up of 100 months, 146 (58.8%) patients had no evidence of disease, 11 (4.4%) had a stable lesion after a debulking and 91 (36.6%) had a recurrence.

Groups II, III, IV and V, which included overall the lowest number of patients, had a recurrence rate of 11.7, 40.0, 31.2 and 7.6, respectively. As to Group VI, 27 patients (40.2%) had a progressing disease while 32 patients (47.7%) had a stable lesion and 8 (11.9%) had a remission.

No significant differences in disease progression was found between the six groups.

#### Recurrence after surgical excision

**Fig. 1:**
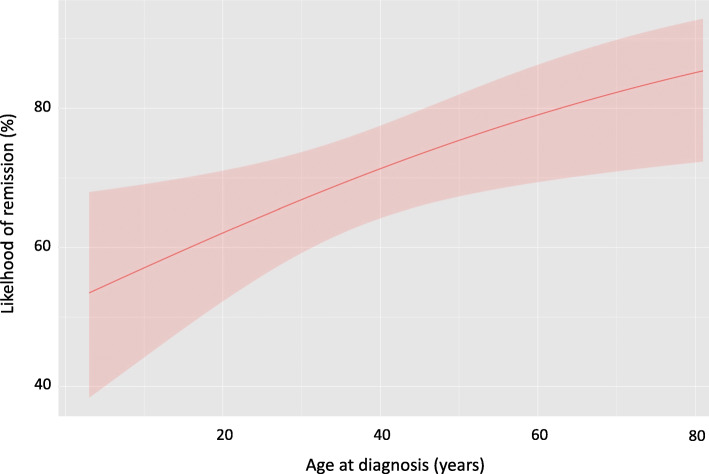
remission likelihood after DTF surgical excision is significantly higher with increasing age (*p* = 0.02). Red line: remission chance. Shaded area: 95% confidence interval

In group I, an older age at diagnosis was associated with a more likely chance of remission (*p* = 0.02), the likelihood of which increased by 2.3% every year (Fig. 1). Moreover, patients with recurrent disease had significantly more recurrences (57.6%) than patients with primary presentation (29.9%) of DTF (*p* = 0.023). Finally, lesions larger than 10 cm had a significantly (*p* = 0.003) lower rate of remission (50.9%) than lesion below 5 cm (65.3%) or in between 5 and 10 cm (59.2%).

#### Progression under active surveillance

In Group VI, patients with a painful lesion had a significant higher (*p* = 0.01) disease progression rate (43.1%) than those who were not in pain (28%). Lesions at the shoulder had significantly higher (*p* = 0.01) progression rate (64.7%) than other anatomic regions.

## Discussion

Firstly, this multicentric study failed to identify significant differences in outcome between all different treatment modalities. Secondly, age was a prognostic factor of outcomes within the surgical resection group as supported by previous studies. Finally, pain was a predictive factor of tumour progression in those patients treated with an ‘active surveillance” approach, which to the best of our knowledge appears to be so far an unpublished finding.

Desmoid fibromatosis of the limbs and girdles is rare but it raises a vivid research and clinical interest because it is locally aggressive and it has a peculiar infiltrative growth which might threaten vital organs, occasionally being described to be fatal as well [17, 19–28, 37]. Furthermore, high recurrence rates in up to two thirds of the patients have been reported independently of the treatment modality [11, 15]. The present study had been conceived to compare different treatment strategies for DTF and to investigate the efficacy of active surveillance policy and the correlation with different prognostic factors either on outcome or on the natural history of the disease.

The study presents limitations: first, this is a multicentric study but, considering the rarity of the disease, we purposed to collect data from different Referrals Centers, on behalf of European Musculo Skeletal Oncology Society, in order to improve the significance of results. Second, the study is retrospective, based on data collection of patients treated with incomplete data regarding beta catenin and surgical margins. Also, data on how the first management was chosen (i.e. multidisciplinary discussion versus single professional decision) is missing. This study design also meant that we were unable to collect data on functional impairment as it is rarely documented routinely. Thirdly, the main limitation of the study was the inhomogeneous cohorts of patients making difficult to identify a management option as better than the others. More than two thirds of the recruited patients were managed surgically, almost one fifth underwent active surveillance and the remnants received miscellaneous treatments which were not eligible to any statistical inference. Therefore, analysis and interpretation of results focused on understanding possible prognostic factors which may affect the course of DTF management in the two largest groups. Furthermore none of the patients in this investigation received kinase inhibitors which are nowadays being proven to be the most effective solution in controlling disease progression [36]. Nevertheless our investigation achieved interesting results with new and original information which will contribute to better understand this rare condition.

Younger patients were found to be at higher risk of recurrence after surgical excision of DTF lesions. This was also observed by other Authors who found that having a DTF excised in the first three or four decades of life, exposes the patient to a significant higher risk or recurrence. Salas et al., in a large multicentric series involving more than 450 patients, observed that people younger than 37 years old had a significant higher recurrence rate than older ones (43% vs 29%) [41]. Similar conclusions have been achieved also by single Institution series, like the one from Massachussets General Hospital in 1998, in which Spear and coworkers found that patients younger than 18 were at higher risk or recurrence after surgical excision [28].

Our study confirmed the existing knowledge that primary DTF lesions have a lower chance of recurrence after surgical excision. In fact, data collected from the EMSOS study showed that non-primary lesions had a recurrence rate which was almost double the figure of primary ones. Spear et al. reported that non-primary tumors had a 77% recurrence rate after surgery compared to 48% of primary lesions [28]. Similarly, Gronchi et al. reviewed the outcome of surgically excised DTF lesions at a single Institution and found that tumor presentation was the strongest factor affecting outcome in a multivariate analysis with non-primary disease having a 17% higher recurrence rate [22].

In our cohort of patients the strongest factor affecting outcome after surgery in a multivariate analysis, was tumor size larger than 10 cm. We recorded that one out of two patients with a lesion larger that 10 cm is likely to experience a recurrence. The same threshold was found to be significant by Crago et al., from Memorial Sloan-Kettering Cancer Center, in a large series including 495 patients with both intra and extra-abdominal fibromatosis [42]. On the other hand, other Authors found smaller lesions, larger than 4-5 cm, to be significantly at high risk of recurrence as well [17, 22].

Differently from other reports, female gender was not found to be a risk factor for local recurrence [43]. Despite DTF is clearly more frequently occurring in females, this does not mean that female are at higher risk of recurrence after surgical excision as confirmed also by other investigators [21, 22, 28]. Deep subfascial location of the lesion did not lead to a higher incidence of failures after surgery as well. Differently from soft tissue sarcomas which are known to be more aggressive when deep, DTF behavior does not change when originating underneath the fascia [4]. Similarly, in our series, the quality of margins did not affect outcome and this is in disagreement with part of the literature, demonstrating that negative margins ensure higher chances of local control. Provided that also other Authors found that the quality of margins is irrelevant on the outcome, our findings are limited by the fact that margins were not assessed in all the cases [4]. Finally, in our series of surgically treated patients, we were not able to identify high risk sites as other Authors did [4, 43].

One fifth of the patients in our series (Group VI) underwent active surveillance. Forty per cent of the patients in this group experienced disease progression, which is similar to 36.6% recurrence rate after surgical excision. At the same time, it has been interestingly found that patients with a painful lesion have a higher risk of disease progression. To our knowledge this is the first report in the literature with such a finding, since pain has not been investigated as a prognostic factor so far.

In particular, pain can be considered a distinctive feature of the natural history of the disease and it might be hypothesized that a painful lesion is active and growing and possibly deserving a different management from painless ones. On the other hand, painful lesions were not found to have a higher failure rate in the surgical group (Group I) and it might be speculated that a painful lesion could be safely surgically excised prior that it reaches a critical size. Then, painful growing lesion biology understanding could add useful information to approach such tumors also non-surgically with medical therapy.

The Desmoid Tumor Working Group has recently published the outcome of the 2018 consensus conference where it has been agreed that active surveillance should be the first line management of desmoid tumors [37]. In the proposed guideline any disease progression should be addressed in first instance with medical therapy with the exception for abdominal wall locations where surgery is indicated. It might be then appropriate, based on our findings, to include in the guideline pain onset as a criterion to label a lesion as possibly progressing and thus necessitating earlier revaluation.

The last relevant finding of this investigation was the greater tendency to progress of the lesions located at the shoulder girdle in the active surveillance group. Other Authors who investigated DTF conservative management found that some sites are at higher risk than others. Bonvalot et al. found limbs location to be more at risk than trunk and neck. In our study we deliberately investigated anatomic locations in more detail and the shoulder girdle resulted to be a hot site for DTF progression [29].

Then, lesions around the shoulder should be either treated or, when observed, the follow-up interval should be very short in order to avoid progression to a threatening size.

In conclusion, although DTF has been described a long time ago, it is still a hot and challenging topic because of local aggressiveness and high recurrence rate, which made difficult to achieve a consensus on the best management, so far. Local recurrence rate after surgery is similar to the observed progression after active surveillance. For this reason, active surveillance in DTF seems to be justified, considering surgery only in selected cases when medical treatment has failed as well, in accordance with the latest guidelines [37]. The present multicentric study, besides confirming already known conclusions on prognostic factors of surgically treated lesions, interestingly highlighted the importance of pain in the natural history of DTF and should then be of inspiration for further research, possibly leading to its optimal management.

## Data Availability

The datasets used and/or analyzed during the current study are available from the corresponding author on reasonable request.
